# It Was Heartbreaking . . . It Was Horrible: The Experiences of Nursing Home Residents’ Children During COVID-19

**DOI:** 10.1093/geroni/igab046.356

**Published:** 2021-12-17

**Authors:** Vivian Miller, Nancy Kusmaul, Jacquelyn Burns, Ji Hyang Cheon

**Affiliations:** 1 Bowling Green State University, Bowling Green, Ohio, United States; 2 University of Maryland, Baltimore County, Baltimore , Maryland, United States; 3 University of Maryland, Baltimore County, University of Maryland Baltimore County, Maryland, United States

## Abstract

As a result of COVID-19, the Centers for Medicare and Medicaid Services (CMS) suspended all outside visitors from entering nursing homes on March 13, 2020. For more than six months, care partners were only permitted in compassionate care situations, so adult children of residents were only able to contact their parents via phone calls, video chats, window visits, and in some cases, limited outdoor visits. Experts have written on the adverse, detrimental impact of this lack of connection and isolation has had on residents. However, the lived experiences from the perspectives of residents’ adult children remain largely absent from the literature. To uncover the experiences of these care partners, semi-structured interviews were conducted (N=12) from December 2020 to February 2021. Adult children shared witnessing their parent’ physical and cognitive decline which they attributed to the lack of visitors. Care partners expressed feeling frustrated that they were unable to observe their parent’s health condition, and could not provide support. Also, many rightfully worried they would never see their loved one again. Findings from this study reveal implications for nursing home leaders and policymakers, such as building infrastructure and systems that both ensure safety and allow care partners to regularly see their residents in long-term care to avoid the unintended adverse consequences of these policies. Further, findings from this research indicate the need for future programs to mitigate and lessen the long-term consequences this isolation has had on both residents and their adult children.

